# Guava residue in poultry diets: an alternative for enhancing sustainability, sensory quality, and economic viability in slow-growing broilers

**DOI:** 10.1007/s11250-026-04854-9

**Published:** 2026-02-04

**Authors:** Mariana Albuquerque Melo, Cláudia Goulart de Abreu, Silvana Cavalcante Bastos Leite, Ana Sancha Malveira Batista, Daiane Félix Santiago Mesquita, Janete Gouveia de Souza, Kélya Jamilha Braga, Robson Mateus Freitas Silveira

**Affiliations:** 1 Agricultural and Biological Sciences Center, Vale do Acaraú State University (UVA), Sobral, CE 62040-370 Brazil; 2 Federal University of Rio Grande do Norte (UFRN), Postal Box 07 - RN 160, Km 03, Jundiaí District, Macaíba, RN 59.280-000 Brazil; 3 Livestock Environment Research Group (NUPEA), Department of Animal Science, “Luiz de Queiroz” College of Agriculture (ESALQ), University of São Paulo (USP), Piracicaba, SP 13418-900 Brazil

**Keywords:** Alternative feed, Alternative poultry farming, *Psidium guajava*, Sensory analysis, Profitability

## Abstract

The valorization of agro-industrial by-products as alternative feed ingredients represents a key strategy for advancing sustainable poultry production within a circular agriculture framework. This study evaluated the effects of partially replacing commercial feed with guava residue (GR) on growth performance, carcass traits, meat sensory quality, organ biometrics, and economic indicators in slow-growing broilers. Two independent trials were conducted: a starter phase (7–28 days, 250 birds) and a grower phase (29–63 days, 200 birds), with diets containing 0, 5, 10, 15, or 20% GR. No performance differences were detected in the starter phase. In the grower phase, 20% GR increased feed intake without affecting body weight, weight gain, or feed conversion. Carcass yield improved at 20% GR, and thigh yield peaked at 5% GR. GR inclusion increased gizzard weight and reduced relative large intestine weight. Sensory analysis indicated an improvement in the colour and aroma of breast meat at higher GR levels (above 10%). Economically, GR reduced feed costs and increased both gross margin and profitability index across input price scenarios. These results demonstrate that guava residue can be included at up to 20% in slow-growing broiler diets to improve meat sensory attributes, enhance profitability, and promote sustainable feed practices aligned with circular agriculture principles.

## Introduction

The Brazilian poultry industry is a global leader, ranking as the world’s third-largest producer and the leading exporter of chicken meat. In 2024, the country produced 14.97 million tonnes of chicken meat and exported 5.29 million tonnes, representing a 3.0% increase in exports compared to the previous year (ABPA 2025). Although conventional, fast-growing genotypes dominate this sector, a growing market segment demands differentiated products, often associated with specific genotypes like slow-growing broilers (SGB) and sustainable feeding strategies.

Slow-growing broilers are typically defined as those with an average daily gain (ADG) of less than 50 g/day, contrasting with fast-growing broilers (FGB), which can exceed 60 g/day (Nicol et al. 2024). These SGB genotypes, such as the commercially available Hubbard Premium Mesclado (Globoaves 2023) strain used in this study, are often selected for alternative production systems due to their distinct physiological traits. Compared to FGB, SGB possess a longer rearing cycle to reach slaughter weight and often exhibit improved leg health and disease resistance (Çapar Akyüz et al. 2022). A key characteristic of these genotypes is their suggested greater resilience to nutritional fluctuations. Studies indicate that SGB are less negatively impacted by suboptimal diets, such as those with lower crude protein levels, compared to their fast-growing counterparts, which suffer significant reductions in growth performance and efficiency under such conditions (Chodová et al. 2021). This inherent robustness opens the door for the use of alternative, less refined feed ingredients without catastrophic drops in productivity.

The high cost of feed, which constitutes 70–75% of total production expenses, is a major constraint, especially for small-scale producers (Valentim et al. 2021). The volatility of conventional feed ingredient prices further incentivises the search for sustainable and cost-effective alternatives (Rufino et al. 2017). In this context, the utilisation of agro-industrial by-products emerges not only as an economic strategy but also an environmental one.

Among various by-products, guava (*Psidium guajava*) residue stands out due to the widespread cultivation of this fruit in tropical and subtropical regions, including South American countries as well as nations such as India, Pakistan, Bangladesh, and Indonesia (Kumar et al. 2022), which confers its valorisation a global relevance. The industrial processing of guava generates a residue that represents approximately 30% of the total fruit volume (Angulo-López et al. 2021). The inadequate disposal of this material generates negative environmental impacts, including methane emissions in landfills and contamination of soils and water bodies. Therefore, its valorization in animal feed aligns directly with the United Nations Sustainable Development Goals (SDGs), particularly with SDG 12 (Responsible Consumption and Production), by promoting the reduction of food waste and the efficient use of natural resources, and contributes to SDG 13 (Climate Action), by mitigating greenhouse gas emissions from the decomposition of organic waste.

Guava residue has a relevant nutritional profile but is lower in crude protein and energy compared to standard corn-soybean meal-based feed (Muniz et al. 2020). Its incorporation into poultry feed could therefore reduce environmental impact and feed costs, provided the animals can maintain satisfactory performance.

Although various studies have investigated the use of guava residue in feeding broiler chickens in industrial systems, usually with rigorous nutritional adjustments to ensure dietary requirements are met (El-Deek et al. 2009; Ogega et al. 2022), this strategy often proves unfeasible for smallholders. Generally, these producers use commercial ready-made feeds or formulate rations on the farm by combining corn and soybean meal with nutritional bases purchased from specialized companies. These bases already contain main ingredients such as macrominerals, microminerals, vitamins, and DL-methionine, as producers lack the technical or financial resources to purchase and dose these components separately. Consequently, the introduction of alternative feeds, such as guava residue, becomes limited, as it requires precise nutritional adjustments that are beyond the operational capacity of these production systems.

Therefore, this study tested a simple and practical strategy suitable for small-scale operations: the direct, non-isocaloric and non-isoproteic partial replacement (0, 5, 10, 15, and 20%) of a standard corn-soybean basal diet with guava residue. The central hypothesis is that the inherent resilience of slow-growing broilers will allow them to maintain productivity despite the progressive dilution of dietary nutrients, thereby validating a straightforward method to reduce feed costs and environmental impact through the valorisation of a local agro-industrial by-product, converging with the principles of the circular economy.

## Materials and methods

### Facilities and bird handling

The experiment was conducted in the Poultry Sector of the Experimental Farm of the Vale do Acaraú State University – UVA, located in Sobral-CE, physiographic zone of Sertão Cearense, at 3º36’ South latitude, 40º1” West longitude, altitude of 56 m. The predominant climate in the region is type BShw” (Köppen classification), megathermal, dry, with an average annual temperature of 26 to 28 °C.

The chickens were housed in a masonry shed built in an east-west direction, with 30-cm side walls and galvanized wire mesh from the walls to the ceiling, a blue raffia curtain on the outside, and a clay tile roof. Inside the shed, the experimental units were installed in 1.5 m x 1.0 m boxes made of metal frames and plastic screens, with a concrete floor covered with wood shavings bedding. Each box contained a tubular feeder and a pendulum drinker, and, for the first 7 days, a 60 W incandescent lamp for artificial heating of the chicks. Water and experimental feed were provided ad libitum throughout the experimental period.

For the implementation of Experiment 1, 25 boxes were used, while an additional 15 boxes were allocated for rearing birds during the pre-experimental phase of Experiment 2. Upon completion of Experiment 1 and following data collection, the birds were sold live to local producers. Once the pens were vacated, the birds designated for Experiment 2 were sexed and weighed, allowing for the setup of the second experimental phase.

### Guava residue

Guava residue from the Sabor da Serra Pulp Factory, located in Meruoca, Ceará, was collected in plastic bags and stored in a cold chamber until being taken to the Vale do Acaraú Experimental Farm, where it was laid out on plastic tarps and dried in open field for four days, turning twice a day and covering with plastic tarps at night. After drying, the residue was ground in 4-mm sieves and stored at the feed factory in bags on wooden platforms for later diet mixing. The guava residue used showed in its composition the following levels: 95.8% dry matter (DM), 10.9% crude protein (CP), 3.1% ash (MM), 10% ether extract (EE), 37.0% crude fiber (CF), 52.5% neutral detergent fiber (NDF), and 32.2% acid detergent fiber (ADF), on a fresh matter basis.

### Experimental design, treatments and experimental diets

Two independent experiments were conducted: the first involving birds in the starter phase (7 to 28 days of age), and the second with birds in the grower phase (29 to 63 days of age). The decision to use distinct groups of birds for each phase was intentional, aiming to prevent residual (carryover) effects of the initial diets on subsequent performance, thereby enabling an isolated assessment of the effects of guava residue at each physiological stage. At the end of the starter phase, the first experiment was concluded, and the grower phase experiment was initiated with a new batch of birds.

Both experiments were conducted using birds of the Hubbard Premium Mesclado lineage (Globoaves 2023). In experiment 1 (initial phase), 250 7-day-old, unsexed chicks with an average weight of 100.2 g ± 0.94 were distributed in a completely randomized design, with 5 treatments, 5 replicates and 10 birds per replicate.

In Experiment 2, sex was used as the blocking criterion in a randomised block design. This approach was adopted to statistically control for the known variation in performance between males and females at more advanced ages, thereby enhancing the precision of the experiment in detecting the effects of dietary treatments. Two hundred 29-day-old birds were used, 80 females, with an average weight of 768.1 g ± 8.6 and 120 males, with an average weight of 842.4 g ± 10.5, distributed in a randomized block design, with 5 treatments, with 5 replications (2 replications in the female block and 3 replications in the male block), and 8 birds per replication.

The treatments consisted of a control diet and four diets with increasing levels of feed replacement by guava residue (5, 10, 15, and 20%). The control diets used in the experiments in the starter and grower phases were formulated to meet the nutritional requirements of slow-growing chickens recommended by Figueiredo (2022) and the ingredient composition values according to Rostagno et al. (2017) (Table 1).

**Table 1:** Percentage and nutritional composition of experimental diets for slow-growing chickens

Ingredients	Initial phase					Growth phase				
	0%	5%	10%	15%	20%	0%	5%	10%	15%	20%
Corn grain	63.523	60.347	57.171	53.995	50.818	69.063	65.610	62.157	58.704	55.250
Soybean meal 46% CP	32.846	31.204	29.561	27.919	26.277	27.390	26.021	24.651	23.282	21.912
Guava residue	0.000	5.000	10.000	15.000	20.000	0.000	5.000	10.000	15.000	20.000
Dicalcium phosphate	1.963	1.865	1.767	1.669	1.570	1.824	1.733	1.642	1.550	1.459
Limestone	1.024	0.973	0.922	0.870	0.819	1.160	1.102	1.044	0.986	0.928
Common salt	0.407	0.387	0.366	0.346	0.326	0.330	0.314	0.297	0.281	0.264
Vitamin Premix ^1^	0.125	0.119	0.113	0.106	0.100	0.125	0.119	0.113	0.106	0.100
Mineral Premix ^2^	0.063	0.060	0.057	0.054	0.050	0.063	0.060	0.057	0.054	0.050
DL-Methionine	0.049	0.047	0.044	0.042	0.039	0.045	0.043	0.041	0.038	0.036
Total	100.00	100.00	100.00	100.00	100.00	100.00	100.00	100.00	100.00	100.00
Metabolizable energy (kcal/kg)	2,920	2,850	2,781	2,711	2,641	2,980	2,907	2,835	2,762	2,689
Crude protein (%)	20.3	19.8	19.4	18.9	18.4	18.2	17.837	17.474	17.111	16.748
Calcium (%)	1.00	0.953	0.905	0.858	0.810	0.99	0.943	0.896	0.849	0.802
Available phosphorus (%)	0.47	0.448	0.426	0.404	0.382	0.43	0.410	0.390	0.370	0.350
Sodium (%)	0.18	0.171	0.162	0.153	0.144	0.16	0.152	0.144	0.136	0.128
Methionine + cysteine (%)	0.71	0.701	0.692	0.683	0.674	0.65	0.644	0.638	0.632	0.626
Lysine (%)	1.10	1.054	1.007	0.961	0.914	0.93	0.892	0.854	0.816	0.778
NDF (%)	13.43	15.38	17.34	19.29	21.25	13.40	15.36	17.31	19.27	21.22
ADF (%)	4.57	5.95	7.33	8.71	10.09	4.34	5.73	7.12	8.51	9.90

^1^ Rovimix - Guaranteed levels per kilogram of product: vitamin A, 12,000,000 IU; vitamin D3, 2,500,000 IU; vitamin E, 30,000 IU; vitamin B1, 2 g; vitamin B6, 3 g; calcium pantothenate, 10 g; biotin, 0.07 g; vitamin K3, 3 g; folic acid, 1 g; nicotinic acid, 35 g; zinc bacitracin, 10 g; choline chloride, 100 g; vitamin B12, 15,000 mcg; selenium, 0.12 g; ^2^ Roligomix - Guaranteed levels per kilogram of product: manganese, 160 g; iron, 100 g; zinc, 100 g; copper, 20 g; cobalt, 2 g; iodine, 2 g.; NDF: Neutral detergent fiber; ADF: Acid Detergent Fiber

During the pre-experimental period (days 1 to 7 in Experiment 1 and days 1 to 28 in Experiment 2), the birds received the same diet, formulated identically to the control diet used in the starter phase.

### Variables evaluated

#### Performance

Feed intake (FI), body weight (BW), weight gain (WG), and feed conversion rate (FCR) were assessed. Birds and feed were weighed at the beginning and end of each experimental phase. In the event of mortality, leftover feed and dead birds were weighed to adjust for performance variables.

#### Carcass and meat cut yield, and organ biometrics

At 70 days, two birds per replicate, which were representative of the plot’s average weight (± 10%), were selected. These birds were then fasted for 8 h, weighed to obtain fasting body weight, and euthanized by cervical dislocation (CONCEA, Normative Resolution No. 37, February 15, 2018). After verifying stunning and unconsciousness, bleeding was performed for three minutes, followed by scalding, plucking, and evisceration. The cleaned and eviscerated carcasses, without feet and head, were weighed to obtain carcass yield (in relation to fasting body weight). The yields of the breast, thigh, drumstick, and wing were calculated in relation to the eviscerated carcass weight (without feet and head), and the relative weights of the heart, liver, gizzard, small intestine, large intestine, and abdominal fat were calculated in relation to fasting body weight .

#### Sensory analysis

Sensory analysis was conducted to assess overall acceptability and sensory quality as perceived by the consumer (Stone et al. 2020). The study was carried out at the Agricultural Products Technology Laboratory (TPA) at Vale do Acaraú State University. The night before analysis, the meat samples were slowly thawed in a refrigerator at 5 °C. Then, 2-cm cubes were obtained and cooked on an electric griddle at 180 °C for 6 min per side, until reaching an internal temperature of 72 °C ± 1 °C, monitored with a skewer thermometer. The samples were maintained at 55 °C until serving time. A panel of 90 untrained consumers was recruited, comprising 58 females and 32 males, with the predominant age range being between 21 and 30 years. The use of a consumer panel is a well-established method for the affective evaluation of acceptability and is considered more suitable for predicting market preference than trained panels when the objective is to measure the perception and liking of the end consumer (Cabrol et al. 2022; Tura et al. 2024). The volunteers confirmed they had no sensory impairments and were not allergic to food of animal origin. The following parameters were evaluated: colour, aroma, flavour, softness, juiciness and overall preference, using a nine-point hedonic scale (1 – “Disliked very much” to 9 – “Liked very much”), in accordance with the methodology used to obtain product acceptance results (Dutcosky 2011). Between samples, panellists were instructed to cleanse their palates with water and unsalted crackers.

#### Economic analysis - partial economic results

The economic assessment of an agricultural activity is based on its profitability, which can be estimated through the indicators described below (Campos 2013):

- Gross Income:

Gross Income corresponds to the total revenue obtained from the sale of products generated by the activity. It is calculated as:

1 $$\:GI=\sum\:_{i=1}^{n}\left(PiQi\right)$$

where:

GI = gross income from production (i.e., sale of live chickens);

Pi = producer price of product i, (i = 1,2, n);

Qi = quantity produced of product i.

b) Total Variable Cost (TVC):

Total Variable Cost (TVC), also referred to as Effective Operating Cost (EOC), represents expenses directly linked to production. In this study, TVC was considered equivalent to Feed Cost (FC), calculated by multiplying feed intake per bird (FI, kg/bird) by the Experimental Feed Cost (EFC), based on ingredient prices in the Sobral region.

Costs were computed for each rearing phase (7–28 days and 29–63 days), and the pre-experimental feed cost (1–6 days; 84 g/bird × basal feed price) was added equally across treatments. The cost of guava residue was estimated by summing transportation expenses (71.2 km round trip, fuel consumption of 10 km/L, gasoline price) and labor costs (four hours of work for receiving, drying, turning, shredding, and bagging). This total was divided by the yield of dried, shredded residue (260 kg). Ingredient prices were then multiplied by their inclusion levels to determine the EFC.

#### Profitability indicators

Profitability indicators assess the activity’s capacity to remunerate production factors (land, capital, labor) and its economic viability in the short, medium, and long term.

#### Gross margin (GM)

Gross Margin (GM) represents the portion of revenue remaining after covering variable (feed-related) costs and indicates whether the activity can meet out-of-pocket expenses. In this study, average values of GI and TVC were used to compute the average GM. It is calculated as:

2 $$GM{\text{ }} = {\text{ }}GI{\text{ }} - {\text{ }}TVC$$

where:

GM = gross margin average;

GI = gross income from production;

TVC = total variable cost;

#### Profitability index (PI)

The Profitability Index demonstrates the rate of return on capital employed, considering only feed-related costs. This indicator measures the return obtained for each real invested in feed. It is expressed as:

3 $$\:PI\:=\frac{GM}{TVC}\:X100$$

#### Scenario analysis

Profitability was evaluated under two scenarios based on fluctuations in input prices:

- Scenario 1: Highest increase in corn and soybean meal prices.
- Scenario 2: Highest decrease in these prices.

Weekly prices of these ingredients were monitored from November 2023 to August 2024. Gasoline prices were also tracked to estimate variations in guava residue cost. Additionally, the average hourly wage of rural workers in Ceará (R$9.23/hour) and the average sale price of live free-range chickens (R$40.00) were considered.

### Statistical analysis

The data were initially tabulated in Microsoft Excel and subsequently subjected to statistical analysis in R software. The data were subjected to the Shapiro-Wilk normality test at a 5% probability level. Normally distributed response variables were subjected to analysis of variance, considering the factorial model, and the means of the treatments with guava residue replacement were compared with the control diet using the Tukey test. Non-normally distributed variables were performed using the non-parametric Kruskal–Wallis test to assess the presence of significant differences among treatments. When a significant effect was detected, pairwise comparisons of means were conducted using the Mann–Whitney test with Bonferroni correction. A 5% probability of error was considered in all analyses.

## Results and discussion

### Performance

#### Experiment 1

In the starter phase, there was no effect (*P* > 0.05) of the levels of feed substitution with guava residue on any of the studied performance variables. Similarly, there was no difference (*P* > 0.05) when individually comparing the results obtained at each substitution level with the control diet (Table 2).

**Table 2:** Performance of slow-growing chickens in the initial phases, from 7 to 28 days (Experiment 1), and in the growth phase, from 29 to 63 days (Experiment 2), fed diets containing different levels of guava residue as a replacement for feed

Guava residue (%)	Experiment 1 (7 to 28 days)				Experiment 2 (29 to 63 days)			
	BW (g/bird)	FI (g/bird)	WG (g/bird)	FCR (g/g)	BW (g/bird)	FI (g/bird)	WG (g/bird)	FCR^1^ (g/g)
0	806.9	1317.5	706.7	1.864	2542.6	4083.3 b	1730.1	2.354
5	772.0	1282.9	671.8	1.910	2689.0	4286.9 b	1875.0	2.286
10	795.6	1275.7	695.4	1.834	2625.0	4197.1 b	1812.0	2.316
15	793.6	1285.5	693.4	1.854	2650.8	4408.1 b	1839.4	2.396
20	773.5	1290.8	673.3	1.917	2636.6	4530.6 a	1824.0	2.484
SEM	6.38	7.15	6.34	0.02	22.08	42.54	22.19	0.03
Effect	Probability							
Linear	0.9728	0.6681	0.9731	0.9592	0.5895	0.0565	0.6108	0.0165
Quadratic	0.1789	0.7219	0.1775	0.1090	0.6470	0.3145	0.6661	0.9588

^a, b^ Means followed by different letters differ from the mean of the control diet, according to Dunnett’s test, at 5% probability; BW = Body weight; FI = Feed intake; WG = Weight gain; FCR = Feed conversion rate; SEM = Standard error of the mean;

^1^ Linear effect of feed replacement levels by GR on FCR (Y = 2.2165 + 0.01398x, R² = 0,98)

Guava residue has 10.9% CP and a high NDF content (52.5%) in its composition, which limits its energy utilisation, estimated at approximately 1527 kcal of ME/kg according to the prediction equation of Nascimento et al. (2009). Based on these parameters, at the highest level of feed substitution (20% GR), there was a 9.6% reduction in the supply of ME (from 2920 to 2641 kcal/kg) and a 9.4% reduction in CP (from 20.3 to 18.4% CP) in the starter phase diet (Table 1), as no nutritional correction of the diets was performed.

Given this scenario, an increase in feed intake was expected to compensate for the energy dilution of the diet with GR substitution, but this did not occur, probably due to the physical limitations imposed by the developing gastrointestinal tract (GIT). Young birds have limited gastric capacity, and intake is controlled by physical mechanisms, such as the distension of the crop and gizzard, which signal satiety (Ferket and Germat 2006). Thus, the inclusion of guava residue, an ingredient with high fibre content, may have promoted satiety more rapidly and for a prolonged period, filling the GIT in a way that restricted intake before the energy requirement was fully met. This physical limitation overrides physiological regulation based on energy demand. Furthermore, gut health and integrity are crucial for efficient digestion and absorption (Adedokun and Olojede 2019). Although it was not the main focus, it is possible that the diet with guava residue, within the 20% inclusion level, maintained the integrity of the GIT, allowing the available nutrients to be used efficiently, which explains the maintenance of weight gain without impairments.

Nevertheless, the weight gain and feed conversion ratio of the birds that received GR were similar to those observed in the birds that received the control diet, suggesting that the nutritional requirements were met in this phase. This phenomenon is supported by the scientific literature, which indicates that the response to reduced-protein diets is influenced by the birds’ genotype. Chodová et al. (2021) demonstrated that slow-growing chickens (ISA Dual) did not show significant changes in final weight or daily weight gain when fed a diet with a 7% protein reduction. In contrast, fast-growing (Ross 308) and medium-growing (JA 757) chickens showed significantly inferior performance under the same conditions. The authors concluded that slow-growing birds have a higher tolerance to nutritional restrictions, a finding that directly supports the results observed in the present study. This inherent resilience of slow-growing genotypes to lower nutrient supply explains the maintenance of performance even with the reduction in ME and CP in the diets containing guava residue.

#### Experiment 2

During the growth phase, compared to birds fed the control diet, feed intake was higher (*P* < 0.05) only at the 20% replacement level. There were no significant differences (*P* > 0.05) for body weight, weight gain, and feed conversion ratio when analysed by Dunnett’s test (Table 2).

In this context, the increased intake of the diet containing 20% guava residue (GR) manifested as a compensatory strategy in the face of the reduction in the energy density of the feed, resulting from the high level of substitution with the fibrous by-product. In this phase, the replacement of 20% of the feed with GR reduced the metabolisable energy supply in the diet, falling from 2,920 in the control diet to 2,689 kcal/kg. As an adaptive response, there was an increase in feed intake, resulting in a similar metabolisable energy intake (348 kcal/day) to that of the control diet (347 kcal/day). This adjustment allowed the birds to meet their energy requirements and maintain performance, as evidenced by the lack of a negative effect on weight gain and feed conversion ratio when compared to the control.

These findings are aligned with the glucostatic theory of intake control, in which birds modulate their intake to meet their energy demand. In older birds, which have a more developed gastrointestinal tract with greater capacity, the physical limit imposed by gut fill becomes less restrictive, allowing the birds to compensate for the lower energy density of the diet through a higher intake volume (Ferket and Germat 2006).

However, a more detailed analysis via polynomial regression, considering only the levels with GR inclusion (5% to 20%), revealed a distinct behaviour. Although no linear or quadratic effect (*P* > 0.05) of the replacement levels on feed intake, weight gain, and body weight was detected, a significant increasing linear effect (*P* < 0.05) on feed conversion ratio was observed. This indicates that, as the levels of GR in the diet increased from 5% to 20%, there was a progressive worsening in feed efficiency (Y = 2.2165 + 0.01398X, R² = 0.98), even in the absence of significant changes in intake or weight gain individually.

This apparently contradictory result—where compensatory consumption at the 20% level-maintained weight gain, but the regression analysis revealed a progressive worsening of FCR—can be explained by the combination of two factors: first, the nature of the ingredient itself, which, being more fibrous and less digestible, may have reduced the efficiency of nutrient absorption (Jha and Mishra 2021). Second, diets that require greater digestive work and generate more metabolic heat can be particularly unfavourable for birds in the growth phase, potentially leading to a diversion of nutrients towards maintaining homeostasis at the expense of weight gain. Thus, the increase in feed intake was not able to fully compensate for the poorer nutritional quality of the diet at the higher inclusion levels, progressively impacting feed efficiency negatively.

In our study, for every 5% substitution of the control diet with GR, the NDF content of the diet increased by 2% points, rising from 13.4% in the control diet to 21.4% in the diet with 20% GR, representing a 58% increase in NDF content. GR has higher levels of insoluble fibre in its composition, as most of its content comes from the seeds, which contain about 60% of this fibre, while the percentage of soluble fibre is approximately 5%. However, the guava peel, also present in GR, has a higher content of soluble fibre, about 15%, and insoluble fibre at 40% (Roberto 2012).

According to Sacranie et al. (2012), insoluble fibers exert a positive modulating effect on the digestion of nutrients, as they slow the transit of the feed bolus in the upper portion of the gastrointestinal tract, reducing the speed of passage, promoting greater gastroduodenal reflux and prolonging the exposure of the feed constituents to HCl and proventriculus enzymes, which enhances digestive efficiency.

Goulart et al. (2016) highlight the benefits of moderate amounts of soluble fiber, which, when fermented by the gut microbiota, generates short-chain fatty acids, used as an energy substrate by enterocytes, stimulating the development of the intestinal mucosa. At the same time, the fermentation of these fibers reduces intestinal pH, creating an environment conducive to the selective proliferation of beneficial bacteria over pathogenic ones, improving gut health.

Lira et al. (2013), using GR at up to 12% inclusion in diets for industrial broiler chickens from 1 to 42 days of age, and Vieira et al. (2023), working with up to 15% GR in diets for slow-growing chickens, also found no effect of inclusion levels on bird performance characteristics. However, unlike the present study, the diets used by these authors were isoenergetic and isonutritive.

Thus, although the technical feasibility of including up to 20% of GR in the diet has been proven, the negative effects on feed conversion highlight the importance of a more detailed economic analysis. The assessment should consider the cost of GR, which is generally lower compared to conventional feed, and its impact on the total cost of feeding. As observed in studies of alternative feeding systems (Souza et al. 2024), the reduction in the cost per kilogram of feed can partially offset the adverse effects on feed conversion, provided the residue is obtained in an accessible and sustainable manner.

The successful incorporation of up to 20% guava residue without major performance impairments underscores the importance of matching alternative feeding strategies with the appropriate animal genotype. The resilience of slow-growing chickens is twofold: they are not only more tolerant of diets with lower protein and energy density (Chodová et al. 2021) but are also better equipped to handle the associated increase in metabolic heat due to their inherently lower heat production (Karaarslan and Nazlıgül 2024). This dual aptitude makes them uniquely suited for sustainable production systems.

### Carcass composition and meat quality

With the levels of 20% and 5% of GR replacing the feed, there was a higher carcass and thigh yield (*P* < 0.05), respectively, in relation to the values obtained with the control diet, with no effect (*P* > 0.05) on the yield of other cuts. A decreasing linear effect (*P* < 0.05) of the feed replacement levels by guava residue was also observed for thigh yield (Y = 18.25–0.13X, R ^2^ = 0.74), indicating a better yield of this cut with the use of 5% of GR in the diet (Table 3).

**Table 3:** Yield (%) of carcass and cuts, relative weight (%) of organs and abdominal fat of slow-growing chickens, according to different levels of guava residue replacing feed in the 29 to 63-day-old phase (Experiment 2)

Guava residue (%)	Carcass	Chest	Thigh	Drumstick	Wing	Heart	Liver	Gizzard	Small intestine	Large intestine	Abdominal fat
0	71.4	28.8	15.6	15.8 b	12.1	0.53	1.33	1.40 b	2.40	0.58	1.46
5	75.8	30.0	14.7	17.8 a	11.7	0.52	1.40	1.73 a	2.58	0.70	1.58
10	75.5	28.5	15.4	17.0 b	12.1	0.52	1.42	1.90 a	2.40	0.71	1.94
15	74.7	29.9	15.2	15.6 b	11.7	0.54	1.53	1.85 a	2.59	0.65	2.00
20	77.0*	30.4	14.7	16.1 b	11.9	0.50	1.42	1.86 a	2.39	0.57	1.62
SEM	0.63	0.31	0.14	0.24	0.06	0.52	1.42	1.75	2.47	0.64	1.72
Regression	Probability										
Linear	0.4853	0.3956	0.9885	0.0248	0.7686	0.6740	0.4694	0.3882	0.3469	0.0218	0.8655
Quadratic	0.1558	0.1426	0.0910	0.2437	0.6069	0.2834	0.1861	0.2807	0.8860	0.3036	0.0899

^a, b^ Means followed by different letters differ from the mean of the control diet, according to Dunnett’s test, at 5% probability; SEM = Standard error of the mean

When compared with the control diet, there was no difference (*P* > 0.05) in the relative weights of the heart, small intestine, large intestine, and abdominal fat. For the gizzard, all diets with GR inclusion showed higher relative weights (*P* < 0.05) than those observed in birds with the control diet (Table 3). Lira et al. (2009) reported a linear increase in gizzard weight of broilers with increasing guava residue inclusion levels of 3%, 6%, 9%, and 12%. However, this effect was not observed in the present experiment.

Lira et al. (2009) found no effect of inclusion levels on the weights and yield of breast, thigh, and drumstick of chickens fed diets with 3, 6, 9, and 12% guava residue. However, the authors report that relative wing weight decreased linearly with inclusion levels. Vieira et al. (2023) reported no differences in carcass and cut yield as a function of dietary GR levels, which varied from 0, 5, 10, and 15%, but found an increase in gizzard weight with increasing GR levels.

As highlighted by Shang et al. (2020), the influence of feeding on gizzard characteristics is associated with mechanical stimulation of this organ, which depends on the level, type of ingredient, size, and characteristics of the feed particles. The more stimulated the mechanical activity, the greater the gizzard weight. In the present study, the inclusion of GR in the diets resulted in greater gizzard weight compared to the control diet.

This effect can be attributed to the high fiber content present in guava residue, whose main fiber components are cellulose, an insoluble fiber, and pectin, a soluble fiber (Kamal et al. 2023). Both types play a fundamental role in the functional dynamics of the gizzard: cellulose, by remaining retained for longer in the upper portion of the gastrointestinal tract, intensifies mechanical stimulation, promoting the strengthening of the muscular layers of this organ (Kiarie et al. 2014); pectin, by increasing the viscosity of the digesta, reduces the rate of passage of food, prolonging its residence in the gizzard and optimizing the digestibility of nutrients (Tejeda and Kim 2021).

According to Abdollahi et al. (2019), this increase in gizzard weight is a typical adaptive response in birds fed fibrous diets and may be beneficial in more sustainable production systems. Thus, the increased fiber content resulting from the replacement of the control diet with the RG diet may have favored prolonged food retention in this organ, stimulating its motility and enhancing its muscle development.

In the large intestine, there was a decreasing linear effect (*P* < 0.05) of the levels of diet replacement with guava residue on relative weight (Y = 0.77–0.009X, R ^2^ = 0.82). These data suggest that increasing levels of GR in the diet are associated with a reduction in large intestine development, which may be related to changes in the fermentation pattern or nutrient digestibility. The reduction in large intestine weight may indicate a direct impact on energy metabolism and the utilization of fiber present in guava residue. Previous studies (Singh and Kim 2021) suggest that diets high in soluble fiber, such as that found in fruit residue, may alter the gut microbiota and reduce the need for large intestine development due to the reduced fermentation requirement.

Although other organs, such as the heart and liver, did not show significant changes in relation to the inclusion of guava residue, the stability of these parameters suggests that partial replacement of the feed with the residue does not compromise the systemic functionality of the birds. These findings are consistent with the results of Camelo et al. (2015), who found that the inclusion of certain agricultural byproducts, such as guava, and, as evidenced by Vieira et al. (2021), cassava, did not compromise the integrity of the organs, at different inclusion levels.

The diet influenced (*P* < 0.05) the color and aroma of the meat of chickens fed guava residue (Table 4), with an increase in the intensity of these characteristics being observed as the levels increased. The diet did not interfere with flavor, tenderness, juiciness and preference (*P* > 0.05).

**Table 4:** Sensory characteristics of meat from slow-growing chickens fed diets according to different levels of guava residue replacing feed in the 29 to 63-day-old phase (Experiment 2)

Guava residue (% )	Colour	Aroma	Flavour	Softness	Succulence	Preference
0	4.64 c	6.07 b	6.42	7.20	6.70	6.13
5	5.87 b	6.20 b	6.27	7.02	6.50	6.06
10	7.28 a	6.77 a	6.76	7.36	6.59	6.88
15	7.52 a	7.20 a	6.68	7.45	6.90	6.75
20	7.51 a	7.06 a	6.35	7.02	6.75	6.34
Mean	6.56	6.66	6.5	7.21	6.69	6.43
SEM	0.37	0.31	0.36	0.33	0.34	0.39
Probabilities	< 0.0001	< 0.0001	0.3954	0.3954	0.3538	0.0585

^a, b^ Means followed by different letters in the row differ significantly according to the Mann–Whitney test with Bonferroni adjustment at the 5% significance level; SEM: Standard Error of the Mean

Replacing guava residue in the diet of slow-growing chickens at levels of 10, 15, and 20% resulted in more intense meat color (*P* < 0.05). Guava is rich in carotenoids and lycopene, which have pigmenting properties and are responsible for the yellow/orange and red hues, respectively (Pestana et al. 2020), which may have contributed to improving the color of the chicken meat.

Guava residue above 10% replacing the basal diet improved the aroma of chicken meat (*P* < 0.05). It is plausible that this effect is mediated by the transfer and metabolism of the numerous volatile compounds present in guava. The literature reports that the fruit’s distinctive aroma is attributed to a complex mixture of compounds, with a quantitative predominance of C6 aldehydes, such as hexanal and (E)-hex-2-enal, alongside other components like limonene, β-caryophyllene, and various esters (Idstein and Schreier 1985; Quijano and Pino 2007). Thus, it is possible that a combination of these compounds, rather than a single substance, acted synergistically to enhance the meat’s aroma.

### Economic assessment of GR replacement in poultry diets

There was significant variation in input prices throughout the months of the study (Table 5). The highest price observed for soybean meal (R$3.38/kg) occurred in November 2023. The lowest price (R$2.38/kg) was observed in April 2024, representing a 29.5% decrease compared to the highest price during the study period. For corn, the highest price observed (R$1.50/kg) was in October 2023, and the lowest price (R$1.18/kg) was observed in July 2024, representing a 21.3% decrease.

**Table 5:** Minimum and maximum monthly prices of inputs practiced in Sobral - CE in the period from November 2023 to September 2024

Month/year	Soybean meal (*R*$/kg)		Corn (*R*$/kg)		Gasoline (*R*$/liter)	
	Minimum	Maximum	Minimum	Maximum	Minimum	Maximum
Oct/2023	2.90	2.90	1.50	1.50	5.69	6.09
Nov/2023	3.13	3.38	1.26	1.30	6.04	6.14
Dec/2023	3.17	3.31	1.37	1.44	5.97	6.17
Jan/2024	2.61	3.17	1.32	1.48	5.89	6.09
Feb/2024	2.50	2.50	1.32	1.34	5.99	6.19
Mar/2024	2.38	2.43	1.31	1.33	5.99	6.19
Apr/2024	2.36	2.43	1.23	1.30	5.99	6.19
May/2024	2.50	2.97	1.22	1.26	6.04	6.24
Jun/2024	2.88	3.04	1.21	1.24	6.04	6.34
Jul/2024	2.70	2.88	1.18	1.25	6.34	6.69
August 2024	2.61	2.70	1.25	1.27	6.49	6.69
Set/2024	2.70	2.74	1.30	1.34	6.49	6.74

It can be seen that soybean meal price volatility was significantly higher than that of corn, influenced primarily by international market dynamics. Between November 2023 and early January 2024, soybean meal prices showed an upward trajectory, followed by a decline starting in January, stabilizing from February until the end of April. From that point on, the price resumed its upward trend, reaching its peak in mid-June. Subsequently, although it experienced a further decline, it was less pronounced, accompanied by slight fluctuations, reaching R$2.70 on September 25, 2024.

Tables 6 and 7 demonstrate the economic benefits of using guava residue (GR) as a partial substitute for conventional feed, both in scenarios of higher and lower input prices. Although these scenarios represent opposite ends of the market, they both converge on the same conclusion: the inclusion of GR reduces feed costs, demonstrating its economic viability regardless of fluctuations in the value of commodities such as corn and soybean meal. This context highlights the importance of nutritional strategies that optimize the cost-benefit ratio in poultry production, mitigating the financial impacts of fluctuations in the sector.

**Table 6:** Economic indices of the production of slow-growing chickens fed different levels of guava residue in the diet, in scenario 1, with the highest increase in input prices

Guava residue (%)	Initial phase (7 to 28 days)			Growth phase (29 to 63 days)			TFC* (*R*$/bird)	GM (*R*$/bird)	PI (%)
	FI (kg/bird)	EFC (*R*$/kg)	FC (*R*$/bird)	FI (kg/bird)	EFC (*R*$/kg)	FC (*R*$/bird)			
0	1.318	2.30	3.04	4.08	2.19	8.93	12.16	27.84	229
5	1.283	2.20	2.83	4.29	2.09	8.98	12.00	28.00	233
10	1.276	2.11	2.69	4.20	2.00	8.40	11.28	28.72	255
15	1.286	2.01	2.58	4.41	1.91	8.41	11.18	28.82	258
20	1.291	1.91	2.46	4.53	1.82	8.23	10.88	29.12	268

GR = Guava residue; FI = Feed intake; EFC = Experimental feed cost; FC = Feed cost; TFC = Total feed cost, GM = Gross margin; PI = Profitability index; *TFC = FC from the pre-experimental phase (84 g/bird × R0.19/bird) + FC from 7 to 28 days + FC from 29 to 63 days

**Table 7:** Economic indices of the production of slow-growing chickens fed different levels of guava residue in the diet, in scenario 2, with the greatest drop in input prices

Guava residue (%)	Initial phase (7 to 28 days)			Growth phase (29 to 63 days)			TFC (*R*$/bird)	GM (*R*$/bird)	PI (%)
	FI (kg/bird)	EFC (*R*$/kg)	FC (*R*$/bird)	FI (kg/bird)	EFC (*R*$/kg)	FC (*R*$/bird)			
0	1.318	1.77	2.33	4.08	1.69	6.91	9.39	30.61	326
5	1.283	1.68	2.16	4.29	1.61	6.89	9.20	30.8	335
10	1.276	1.59	2.03	4.2	1.52	6.39	8.57	31.43	367
15	1.286	1.51	1.94	4.41	1.44	6.34	8.43	31.57	374
20	1.291	1.42	1.83	4.53	1.35	6.14	8.12	31.88	393

GR = Guava residue; FI = Feed intake; EFC = Experimental feed cost; FC = Feed cost; TFC = Total feed cost, GM = Gross margin; PI = Profitability index; *TFC = FC from the pre-experimental phase (84 g/bird × R0.15/bird) + FC from 7 to 28 days + FC from 29 to 63 days

The data indicate that the 20% replacement level was the most economically efficient, as it reduced the cost per kilogram of feed from R$2.30 (control diet) to R$1.91 in the high-input scenario and from R$1.77 (control diet) to R$1.42 in the low-input scenario. This savings directly reflects the reduction in feed costs per bird, even with the slight increase in feed intake observed at higher replacement levels. This behavior is in line with previous studies, such as that by Lana et al. (2020), which demonstrated that the inclusion of by-products in the diet can maintain feed efficiency, even with small changes in feed intake.

The analysis shows that the use of guava residue in the feeding of slow-growth chickens is not only an economically viable alternative, but also represents a strategic solution to mitigate production costs in scenarios of high volatility in the prices of conventional inputs.

In both scenarios, a gradual reduction in EFC, FC, and TFC values was observed, while GM and PI increased as the substitution level increased. These results corroborate the economic prognosis of including agroindustrial residues in the diets of slow-growing chickens, further demonstrating their effectiveness in minimizing the effects of high input prices on the financial sustainability of production.

In Scenario 1, using 20% GR in the diet, the TFC reduction was 10.5% compared to the control diet, while in Scenario 2, this reduction was 13.5%. The PI in Scenario 1 increased by 17% and in Scenario 2 by 20%, with the replacement of the feed with guava residue. These results demonstrate that the substitution strategy remains not only advantageous but even more profitable in a less pressured input market.

The increase in profitability with the 20% replacement of feed with GR demonstrates the production system’s ability to generate a higher financial return per real invested in feed. This finding is crucial for small and medium-sized producers, who often face reduced profit margins due to the volatility of traditional input prices (Angelo 2022).

In the calculations performed in this study, the selling price of chicken was not changed to allow for a better comparison. However, in general, the selling price of products tends to rise during periods of rising input prices, albeit to a lesser extent. Therefore, if producers can purchase feed ingredients during periods of low prices, as long as they have the infrastructure to store them properly and use them during periods of high market prices, and can also sell the chickens at a higher price, they will be able to obtain an even better economic return.

Furthermore, another return not accounted for in this study is environmental, since using guava residue in bird feed provides a clean alternative to discarding it in landfills, where it would be deteriorated by microorganisms producing greenhouse gases.

Within the context of alternative poultry production, where feed constitutes a significant portion of total costs (Fiorilla et al. 2024), the use of agro-industrial by-products such as guava residue is a strategic approach. This practice not only reduces reliance on more expensive conventional ingredients like corn and soybean meal but also serves as a pillar of the circular economy by valorising waste streams that would otherwise be discarded (Ogega et al. 2022), thereby enhancing the economic and environmental sustainability of the production system.

This study contributes significantly to improving technical knowledge about the use of agricultural byproducts in poultry farming, highlighting their potential as nutritional inputs. Furthermore, the findings indicate the need for further research to assess the nutritional and productive impacts under different rearing conditions and replacement levels.

## Conclusions

The inclusion of up to 20% guava residue (GR) in the diet of slow-growing chickens can improve meat colour and aroma and provide a greater economic return. This study confirms that this practice is technically and economically viable, aligning with the principles of sustainability and the circular economy. The valorisation of an agro-industrial by-product that would otherwise be discarded, without significantly compromising zootechnical performance, reinforces the potential of GR as an alternative ingredient in poultry farming.

The success of this strategy is directly related to the resilience of slow-growing genotypes, which exhibit greater tolerance to diets with lower nutrient density and better metabolic adaptation. The synergy between the alternative ingredient and the suitable genetic profile is essential for strengthening more sustainable production systems that are less dependent on conventional inputs. Thus, the use of guava residue represents an intelligent solution to reduce costs, add value to the production chain, and promote a more accessible and environmentally responsible poultry industry, especially for small and medium-sized producers.

## Data Availability

No applicable.

## Code availability

No applicable.
